# Regulation effect of Aspirin Eugenol Ester on blood lipids in Wistar rats with hyperlipidemia

**DOI:** 10.1186/s12917-015-0523-5

**Published:** 2015-08-20

**Authors:** Isam Karam, Ning Ma, Xi-Wang Liu, Shi-Hong Li, Xiao-Jun Kong, Jian-Yong Li, Ya-Jun Yang

**Affiliations:** Key Lab of New Animal Drug Project of Gansu Province; Key Lab of Veterinary Pharmaceutical Development, Ministry of Agriculture, Lanzhou Institute of Husbandry and Pharmaceutical Science of CAAS, No.335, jiangouyan, qilihe district, Lanzhou, 730050 China

**Keywords:** Aspirin eugenol ester (AEE), Hyperlipidemia, High fat diet, Rats

## Abstract

**Background:**

Aspirin eugenol ester (AEE) is a promising drug candidate for treatment of inflammation, pain and fever and prevention of cardiovascular diseases with less side effects. The experiment will be conducted to investigate the efficacy of AEE on curing hyperlipidemia in Wistar rats. The rats were fed with high fat diet (HFD) for 8 weeks to induce hyperlipidemia.

**Results:**

Compared with the model group, the results showed that AEE at 54 mg/kg dosage could significantly decrease the hyperlipidemia indexes including triglyceride (TG), low density lipoprotein (LDL) and total cholesterol (TCH) (*p* < 0.01), increase high density lipoprotein (HDL) (*p* < 0.05) for five weeks drug administration. Meanwhile, simvastatin had same effect on hyperlipidemia indexes such as TG, LDL, TC, but no significant increase in HDL.

**Conclusion:**

AEE was effective against hyperlipidemia and had better anti-hyperlipidemic effect than its component, acetylsalicylic acid (Aspirin, ASA), eugenol and integration of ASA and eugenol. Under the experimental circumstance, the optimal dose of AEE to cure hyperlipidemia is 54 mg/kg for five weeks in Wistar rats.

## Background

Hyperlipidemia is a heterogeneous group of disorders characterized by an excess of lipids in the bloodstream. The concentrations of lipids, such as triglycerides (TG), cholesterol (TCH) and low density lipoprotein (LDL) increase, or the level of high density lipoprotein (HDL) decrease in the blood [1]. Hyperlipidemia is becoming a major health problem in the world recently even in human and companion animal clinic [2, 3].

Acetylsalicylic acid (Aspirin, ASA) has been extended to prevention and treatment of cardiovascular diseases [4–6]. Low-dose ASA (5 mg/kg) can ameliorate hyperlipidemia induced by high-fat diet (HFD) and hyperinsulinemia in Sprague–Dawley rats [7]. However, the side effects of ASA, such as serious gastrointestinal damage, have limited the long time using of this classic drug [8]. Eugenol is the main component of volatile oil extracted from dry alabastrum of *Eugenia Caryophyllata Thumb.* Eugenol has many pharmacological activities, including antibacterial, anti-inflammation, analgesia, analgesia, as well as anti-oxidation [9], antiplatelet aggregation [10], and hyperlipidemia amelioration [11, 12]. However, the disadvantages of irritation and vulnerablity to oxidation have limited the application of eugenol in practice [13, 14].

To overcome the disadvantages of ASA and eugenol, aspirin eugenol ester (AEE) as a novel medicinal compound was designed and synthesized according to the prodrug principles of structure recombination [15]. It is a pale yellow and smellless crystal. The stomach, duodenum and ileum showed an increased mucosa height after high-dose AEE exposure (2000 mg · kg^−1^) in a 15-day oral dose toxicity study [16], this was mainly due to increased height of the villus. The acute toxicity of AEE is less than that of ASA and eugenol, only 0.02 times the toxicity of ASA and 0.27 times the toxicity of eugenol in mice [17]. AEE could be metabolized into ASA and eugenol *in vitro* and *in vivo* [18], and had the effects of anti-inflammation, antipyretic, analgesia, and antioxidant [15, 19]. In addition, the previous research indicated that AEE (50 mg · kg^−1^ and 160 mg · kg^−1^) could reduce the serum TC and TG in rats with standard diet [16].

However, AEE as a promising drug candidate for preventing and treating hyperlipidemia, it is important to characterize its efficacy in rats with HFD. The present study was performed to assess the efficacy of AEE against hyperlipidemia. Meanwhile, this study will provide guidance for the design of further studies and clinical trials of AEE.

## Results

### Animal disease model

In regard to blood lipid levels, there was no significant difference between Group I and Group II in week 4 and 6 (data not shown). So the administration time of HFD was prolonged. At week 8, there were significant increase of TG, LDL and TC and decrease of HDL of model group (*p* < 0.01, seen in Table 1), which confirmed that the hyperlipidemia animal model was successfully established by HFD in the experiment.

**Table 1:** Blood lipids level in Group I and II at the end of 8th week after using HFD diet

Variables	Unit	TG	TCH	HDL	LDL
Group I	mmol/L	0.77 ± 0.42	1.01 ± 0.25	0.55 ± 0.07	0.12 ± 0.08
Group II	mmol/L	1.51 ± 0.38^**^	2.18 ± 0.31^**^	0.44 ± 0.06^**^	0.52 ± 0.08^**^

Note: TG: Triglyceride; HDL: High density lipoprotein; LDL: Low density lipoprotein; TCH: Total cholesterol. ^**^ *P* < 0.01 significant difference from blank group

### Body weight

From the results of body weight before drug administration, blank group had a lower body weight than other groups (*P* < 0.01, seen in Table 2), and this indicated that HFD had a remakable influence on body weight. There was no significant difference among other groups except blank group. After drug administration, the average values of body weight in drug treatment group were less than model group and there was still significant difference between blank and model group (*P* < 0.01). The average body weight values in CMC-Na group were similar with model group.

**Table 2:** Body weights changes of rats before and after drug treatment

Groups	Body weight (g)	
	Before drug treatment	Fifth week after drug treatment
A	276.1 ± 6.4^aa^	330.1 ± 5.6^bb^
B	295.4 ± 7.1	358.8 ± 7.2
C	298.4 ± 5.6	347.3 ± 6.8^b^
D	303.6 ± 7.3	340.8 ± 5.9^bb^
E	299.5 ± 6.9	340.8 ± 8.1 ^bb^
F	301.1 ± 5.9	339.3 ± 6.4 ^bb^
G	305.6 ± 6.9	343.4 ± 5.1 ^bb^
H	292.6 ± 5.3	321.1 ± 5.9 ^bb^
I	306.8 ± 5.9	342.4 ± 7.1 ^bb^
J	302.7 ± 6.7	352.3 ± 6.6

Note: A: blank group, B: model group, C: AEE low dose, D: AEE medium dose, E: AEE high dose, F: integration group (acetylsalicylic acid: eugenol, molar ratio 1:1, 0.11 mmol), G: acetylsalicylic acid group, H: eugenol group, I: simvastatin group, J: CMC-Na group. ^aa^ *P* < 0.01 significant difference from model group before administration. ^b^ *P* < 0.05, ^bb^ *P* < 0.01 significant difference from model group after administration. The time before drug treatment was the end of 8th week after HFD diet was used and fifth week after drug treatment was the end of 13th week after HFD diet was used

### Anti-hyperlipidemic effect

Blood samples were taken for lipids examination after the drugs were given for two weeks, but there was no significant difference among groups (data not shown). So the time was extended for five weeks. From table 3, the difference effects of drugs on hyperlipidemia appeared after AEE was given for five weeks. Meanwhile, there was no statistical difference between model and CMC-Na group, which indicated that CMC-Na has no effect on hyperlipidemia indexes as a vehicle control.

**Table 3:** The blood lipids levels at the end of 13th week (after drugs administration for five weeks, *n* = 10)

Variables	Blank	Model	CMC-Na	Simvastatin	AEE 18	AEE 36	AEE 54	Integration	Aspirin	Eugenol
TG	0.77 ± 0.2^**^	1.65 ± 0.22	1.63 ± 0.34	1.12 ± 0.15^**^	1.43 ± 0.11	1.36 ± 0.16^*^	1.23 ± 0.15^**^	1.35 ± 0.17^*^	1.27 ± 0.15^**^	1.39 ± 0.22
HDL	0.46 ± 0.04	0.52 ± 0.05	0.58 ± 0.02	0.56 ± 0.05	0.55 ± 0.04	0.56 ± 0.03	0.62 ± 0.05^*^	0.61 ± 0.02	0.61 ± 0.05	0.56 ± 0.01
LDL	0.11 ± 0.03^**^	0.54 ± 0.04	0.51 ± 0.05	0.23 ± 0.04^**^	0.31 ± 0.04^**^	0.3 ± 0.03^**^	0.25 ± 0.04^**^	0.31 ± 0.02^**^	0.30 ± 0.04^**^	0.28 ± 0.05^**^
TCH	1.01 ± 0.18^**^	2.49 ± 0.14	2.11 ± 0.38	1.61 ± 0.23^**^	1.82 ± 0.16^**^	1.73 ± 0.21^**^	1.55 ± 0.27^**^	1.77 ± 0.11^**^	1.80 ± 0.10^**^	1.68 ± 0.13^**^

Note: TG: Triglyceride; HDL: High density lipoprotein; LDL: Low density lipoprotein; TCH: Total cholesterol; Integration: acetylsalicylic acid:eugenol (molar ratio 1:1, 0.11 mmol). The unit of TG, HDL, LDL and TC is mmol/L. ^*^ *P* < 0.05 significant difference from model group. ^**^ *P* < 0.01 significant difference from model group

In the blood lipid analysis (seen in Table 3), following five weeks administration of drugs, TG, TC and LDL were significantly decreased in varying degrees in comparison with model group. These changes meant that there were significant differences between the treated groups and the model group at the end of 13th weeks. In regard to TG index, the results in ASA and simvastatin groups were significantly reduced when compared with model group (*p* < 0.01). With the increase of AEE dose, the mean values of TG were decreased which showed that TG values were dose-dependent in AEE groups. Meanwhile, TG values in integration group also showed significant difference from model group (*p* < 0.05). AEE at three different doses, simvastatin, ASA, eugenol and integration of ASA and eugenol reduced significantly levels of LDL and TC (*p <* 0.01) when compared with model group. HDL in model group showed no significant difference from blank group at week 13. Only AEE high dose group significantly increased HDL (*p <* 0.05) when compared with model group.

### Optimal dosage of AEE

**Fig. 1:**
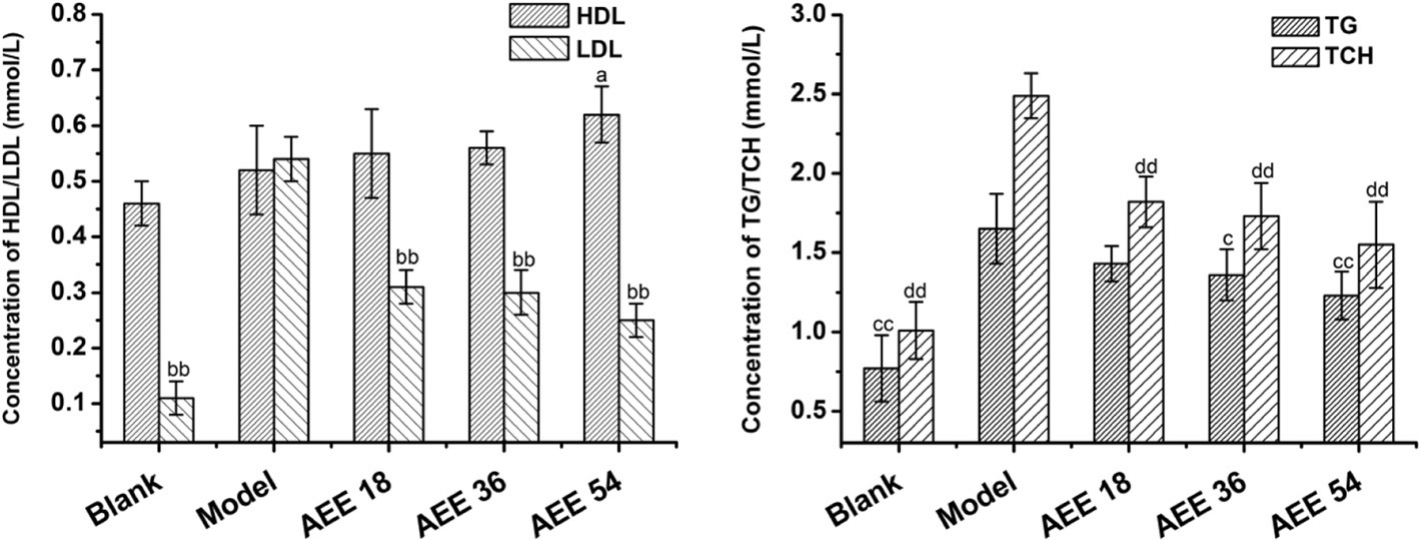
The influence of AEE different dosage on hyperlipemia indexes after administration for five weeks (*n* = 10). TG: Triglyceride; HDL: High density lipoprotein; LDL: Low density lipoprotein; TCH: Total cholesterol. The blank group and AEE groups were used to compare with the model group. ^a^ *P* < 0.05, ^aa^P < 0.01 significant difference of HDL; ^b^ *P* < 0.05, ^bb^ *P* < 0.01 significant difference of LDL; ^c^ *P* < 0.05, ^cc^ *P* < 0.01 significant difference of TG. ^d^ *P* < 0.05, ^dd^ *P* < 0.01 significant difference of TCH. There was no significant difference among three AEE groups in influence on hyperlipidemia indexes

The results demonstrated that AEE had significant effect on ameliorative hyperlipidemia indexes. Through the evaluation of AEE groups, high dose AEE could decrease more significantly the levels of TG, TC, LDL (*p <* 0.01), and at the same time increase HDL level (*p <* 0.05). The effects of AEE at high, medium and low dose on LDL and TC were similar (Figure 1). However, AEE at high dose had more significant effect on TG (*p <* 0.01) and HDL (*p <* 0.05).

### Multiple comparisons

**Fig. 2:**
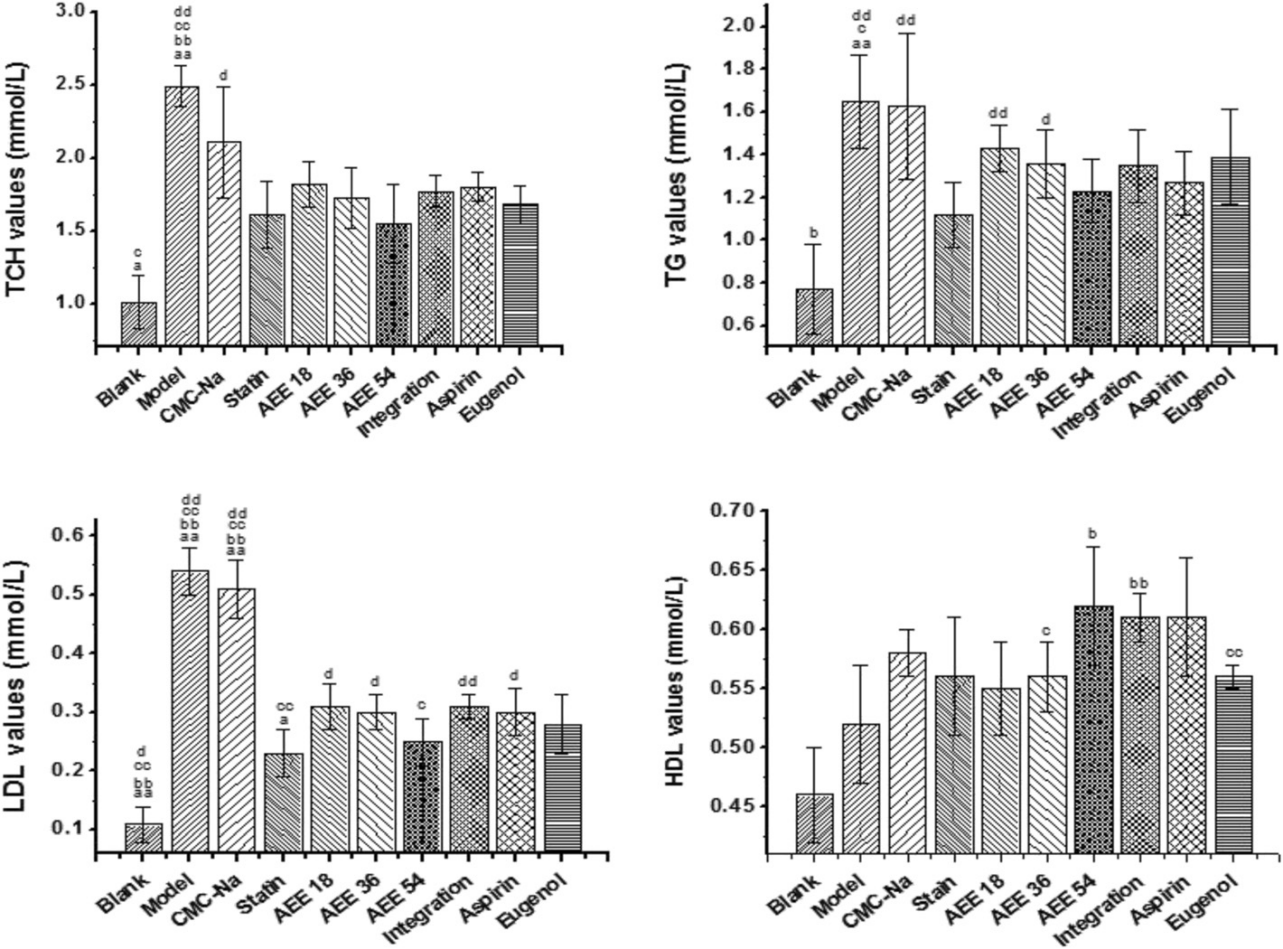
Effects of different drugs on hyperlipemia indexes after drugs administration for five weeks (*n* = 10). TG: Triglyceride; HDL: High density lipoprotein; LDL: Low density lipoprotein; TCH: Total cholesterol. Integration: aspirin and eugenol at the mole ratio 1:1. ^a^ *P* < 0.05 significant difference from aspirin group. ^aa^ *P* < 0.01 significant difference from aspirin group. ^b^ *P* < 0.05 significant difference from eugenol group. ^bb^ *P* < 0.01 significant difference from eugenol group. ^c^ *P* < 0.05 significant difference from integration group. ^cc^ *P* < 0.01 significant difference from integration group. ^d^ *P* < 0.05 significant difference from simvastatin group. ^dd^ *P* < 0.01 significant difference from simvastatin group

Figure 2 showed the different effects of drugs on blood lipid levels. When compared with ASA and eugenol group, there was no significant difference between AEE and other groups, except the HDL index in AEE high dose group (Fig. 2). However, when compared with integration group, there was significant difference of LDL value in AEE high dose group (*p <* 0.05) and HDL value in AEE medium dose group (*p <* 0.05).

Compared with model group, AEE and simvastatin significantly reduced TG, LDL and TC (*p <* 0.01). Meanwhile, AEE increased significantly HDL (*p <* 0.05), but simvastatin had no significant increase of HDL. The results showed that simvastatin had stronger effect than low and medium dose of AEE on TG and LDL value. From the results, there was no statistical difference between simvastatin and AEE high-dose groups, which suggested that the anti-hyperlipidemic effects of high-dose AEE and simvastatin were similar.

## Discussion

Hyperlipidemia, a group of metabolic disorders characterized by the elevated levels of lipids, is a major modifiable risk factor for atherosclerosis and cardiovascular disease [20]. These lipids include cholesterol, cholesterol esters, phospholipids, and triglycerides. Increased levels of LDL are related to the development of atherosclerosis [21, 22]. HDL plays an important role in removing cholesterol from tissues and protecting against cardiovascular disease. Hyperlipidemia can be the result of an inherited disease in certain breeds of dogs [23]. In pets, hyperlipidemia most often occurs as a consequence of some disorder, hyperlipidemia even can also occur spontaneously after a meal of high-fat foods, particularly table scraps [24, 25]. Hyperlipidemia is seen most commonly in ponies, miniature horses, and donkeys, and less frequently in standard-size adult horses [26, 27]. In non-ruminants, including primates and man, hyperlipidemia may be increased by dietary manipulations such as feeding excessive cholesterol or fats with high saturated fatty acid content [28]. A great number of animal models, such pigeons, chickens, swine, cats, dogs, monkeys, mice, rabbits and rats, have been tested [29]. Commonly used rat strains (i.e. Sprague–Dawley, Wistar) typically have high levels of HDL-C and low levels of LDL-C. HFD is capable of promoting elevations in TC and LDL in Sprague–Dawley or Wistar rats likely by reducing bile acid production [30–32].

There are many chemical drugs that could ameliorate hyperlipidemia such as: statins, fibrates, ezetimibe and nicotinic acid, but most of them are expensive and have undesirable effect [33]. So there are increasing interest in alternative drug for the prevention and treatment of hyperlipidemia. Currently available hyperlipidemic drugs have been associated with a number of side effects. Therefore, now it’s important to develop a novel drug that is less toxic, less expensive, which can provide better safety and efficacy on a long term usage.

In this study, the anti-hyperlipidaemic effects of AEE were investigated in rats with induced hyperlipidemia by HFD. The blood lipid indexes were observed during eight weeks. When compared the lipid level between blank and model group at week 8, there was a significant elevation in the levels of serum LDL, TG and TC, and decrease of HDL. Changed levels of these parameters in serum are presumptive markers of hyperlipidemia in serum. LDL is more related to hyperlipidemia and is an important index in these parameters. On other hand, HDL in this experiment decreaseed at week 8 in model group. However, HDL value in model group showed no significant difference from blank group at week 13. This apparently unexpected result could be explained by the light increase of HDL in model group and decrease in blank group, some studies found that the diet content can effect HDL level [34], light decrease of HDL in blank group may due to the feeding long period rich of carbohydrate in standard diet. Some studies found that the increased plasma apoE of apolipoproteins in aged hyperlipidemia rats can increase HDL after a long period [34–38].

From the result in this study, AEE had influence on blood lipid indexes. The changes in TC, TG, LDL and HDL confirmed that AEE had influence on hyperlipidemia. Previous study on AEE showed that it had effect on TG and TC [17]. Compared AEE high dose with simvastatin and CMC-Na, it had positive effect on antihyperlipidemia. AEE high dose significantly decreased TG, LDL, TC and increased HDL index, but simvastatin has no significant increase in HDL, this confirmed that AEE more benefit on curing hyperlipidemia. Based on the results (Table 3), ASA reduced TG significantly meanwhile eugenol had no significant difference on TG, which may speculate that ASA may play a key role on TG reduction from AEE. This may suggest that the effects of simvastatin, AEE and its component on LDL, TC were similar which decreased (*p <* 0.01) compared with model group. On the other hand, CMC-Na as a vehicle had no effect on hyperlipidemia, which confirmed that the influence on hyperlipidemia is due to AEE only.

The previous study showed that eugenol had effect on hyperlipidemia since it is probably mediated through inhibition of hepatic cholesterol biosynthesis, reduction of lipid absorption, enhanced catabolism of LDL-cholesterol, and catabolism of TG [11, 12]. In our study, the results showed that eugenol could reduce significantly the TG and TCH index (Table 3*P* < 0.01). However, there was no statistical difference between eugenol and model group in the TG index. Noteworthy, ASA could reduce TG, LDL and TCH index (Table 3*P* < 0.05), which showed that ASA possessed positive drug effects on hyperlipidemia. The study on the metabolism of AEE showed that AEE was decomposed into ASA and eugenol. Salicylic acid (SA) from AEE metabolism as a final metabolite excreted in urine was coincided with SA from ASA metabolism. However, the elimination time of SA was longer than ASA, which might explain the efficacy of AEE lasting longer than those of ASA and eugenol. Therefore, the AEE effect on hyperlipidemia can come mainly from eugenol and ASA, and the effect of AEE on hyperlipidemia may result from synergetic action of ASA and eugenol together, which could display stronger drug effects than ASA and eugenol.

## Conclusions

In summary, there were significant differences in blood lipid indexes throughout the experimental period under the present study conditions. For Wistar rats, the optimal dose for curing hyperlipidemia was considered to be 54 mg/kg/day administrating for five weeks, further studies should be conducted to investigate its prevention effect and the action mechanism of AEE on antihyperlipidemia.

## Materials and methods

### Chemicals and reagents

ASA eugenol ester (AEE), transparent crystal (purity: 99.5 % with RE-HPLC), was prepared in Key Lab of New Animal Drug Project of Gansu Province, Key Lab of Veterinary Drug Development of Agricultural Ministry, Lanzhou Institute of Husbandry and Pharmaceutical Sciences of CAAS. CMC-Na (carboxyl methyl cellulose sodium) and simvastatin was supplied by Tianjin Chemical Reagent Company (Tianjin, China). ASA and Tween-80 were supplied by Aladdin Industrial Corporation (Shanghai China). Eugenol was supplied by Sinopharm Chemical Reagent Co., Ltd. (Shanghai China). High diet feed (standard rat diet 77.8 %, yolk power 10 %, lard 10 %, cholesterol 2 %, bile salts 0.2 %) consists of 41.5 % lipids, 40.2 % carbohydrates, and 18.3 % proteins (kcal) and standard rat diet consists of 12.3 % lipids, 63.3 % carbohydrates, and 24.4 % proteins (kcal). The feed was supplied from KeaoXieli Co., Ltd (Beijing, China). The TG, TC, LDL and HDL kits were provided by Ningbo Medical System Biotechnology Co., Ltd (Ningbo, China). Erba XL-640 analyzer (German) was used to measure the blood lipid level.

### Animals

Wistar male rats with clean grade, aged 7 weeks and weighing 160–180 g were purchased from the animal breeding facilities of Lanzhou Army General Hospital (Lanzhou, China). They were housed in plastic cages of appropriate size (50 × 35 × 20 cm, ten rats per cage) with stainless steel wire cover and chopped bedding. Light/dark regimen was 12/12 h and living temperature was 22 ± 2 °C with relative humidity of 55 ± 10 %. Rat feed and drinking water were supplied *ad libitum*. The study was performed in compliance with the Guidelines for the care and use of laboratory animals as described in the US National Institutes of Health and approved by Institutional Animal Care and Use Committee of Lanzhou Institute of Husbandry and Pharmaceutical Science of CAAS. Animals were allowed a 2-week quarantine and acclimation period prior to start of the study.

### Drug preparation

AEE, simvastatin and ASA suspension liquids were prepared in 0.5 % of CMC-Na. Eugenol and Tween-80 at the mass ratio of 1:2 mixed with the distilled water.

### Group and dosing

After their arrival for two weeks, rats were randomized into two main groups, group I as blank control (n = 10 rats) feed with basal diet and group II (n = 90 rats) feed with HFD for eight weeks. After hyperlipidemia were induced successfully in rats, the HFD group was averagely divided into nine groups and they were model group and eight treatment groups. So it included eight test groups and two control groups as blank and model groups (seen in Table 4).

**Table 4:** The experimental design in the study

Groups	Number of rats	Food	Drug	Dosage	Average volume per rat
A	10	Basal diet	--	--	--
B	10	HFD diet	--	--	--
C	10	HFD diet	AEE	18 mg/kg	0.58 ml
D	10	HFD diet	AEE	36 mg/kg	1.17 ml
E	10	HFD diet	AEE	54 mg/kg	1.72 ml
F	10	HFD diet	ASA+ Eugenol	(20 + 18) mg/kg	1.19 ml
G	10	HFD diet	ASA	20 mg/kg	0.65 ml
H	10	HFD diet	Eugenol	18 mg/kg	0.54 ml
I	10	HFD diet	Simvastatin	10 mg/kg	0.63 ml
J	10	HFD diet	CMC-Na	20 mg/kg	1.28 ml

Note: HFD: high fat diet. A: blank group, B: model group, C:EE low dose, D: AEE medium dose, E: AEE high dose, F: integration group (ASA: eugenol, molar ratio 1:1), G: acetylsalicylic acid (ASA) group, H: eugenol group, I: simvastatin group, J: CMC-Na group. At the end of 4th, 6th, 8th, 10th and 13th week after HFD was used, the rats were fasted for 10–12 h and anesthetized with 10 % chloral hydrate. Approximately 1.5 ml blood samples were taken from tail tip for blood lipids examination

The dosage for rats was based on individual weekly body weights for five weeks. Drugs were administered intragastrically to each rat. HFD continued during the experiment period.

In order to compare AEE and its precursor, integration of aspirin and eugenol group was designed in the experiment. For the comparability of the results in the experiment, the mole of medium-dose of AEE, aspirin, and eugenol is the same as 0.11 mmol. The integration group administrated both aspirin and eugenol, which were also designed at 0.11 mmol. The 0.5 % of CMC-Na at the dose of 4 ml · kg^−1^ was as the drug vehicle control and the dosage of CMC-Na was close to equal in comparison with AEE and ASA groups. Simvastatin (10 mg · kg^−1^) was choosed as positive control drug to compare with AEE.

### Blood sampling

After fasting for 10–12 h, rats were anesthetized with 10 % chloral hydrate and blood samples were taken from tail tip for blood lipids examination. In order to make sure the volume of the blood sample, the rat tails were immersed in water bath at 45 °C for 5 min to make the blood vessel swelling, then approximately 1.5 ml blood sample was collected from the rat tail. The serums were got through centrifuge for 15 min at the speed of 4000 g at 4 °C.

### Design of the experiment

First hyperlipidemia model disease is needed to be established successfully by HFD. At the beginning of the experiment, the rats were divided into two main groups. Group I feed with standard diet and Group II feed with HFD. The blood samples were taken on 4th, 6th and 8th week to examine blood lipids. After the success of hyperlipidemia animal model, Group II was divided into nine groups, model group and eight treatment groups, and high fat diet was continued during the rest experiment period.

After the administration of drug, blood samples were taken to measure the change in blood lipids on 10th week and 13th week. Blood sample was analyzed with the same method.

### Statistics

The statistical analyses were carried out using IBM SPSS 19.0 (USA). All data obtained from the experiment are expressed as mean ± standard deviation (SD). The difference between Group I and Group II were evaluated by Student’s *t*-Test. Statistical differences between the treatments and the control were evaluated by using one-way ANOVA with Duncan test. *P*-values less than 0.05 were considered statistically significant.
